# Population niche width is driven by within-individual niche expansion and individual specialization in introduced brook trout in mountain lakes

**DOI:** 10.1007/s00442-022-05201-z

**Published:** 2022-06-03

**Authors:** H. K. Baker, C. E. F. Bruggeman, J. B. Shurin

**Affiliations:** Department of Ecology, Behavior, and Evolution, University of California San Diego, La Jolla, CA 92093 USA

**Keywords:** Ecological opportunity, Intraspecific variation, Introduced predators, Niche evolution, Resource use

## Abstract

The width of a population's resource-use niche is determined by individual diet breadth (“within-individual component”) and the degree of niche partitioning between individuals (“between-individual component”). The balance between these two factors affects ecological stability and evolutionary trajectories, and may shift as ecological opportunity permits broader population niches. Lakes in California’s Sierra Nevada Mountains vary in resource diversity for introduced brook trout (*Salvelinus fontinalis*) due to elevation, lake morphometry, and watershed features. We compared the relative contributions of within- and between-individual niche components to two measures of the dietary niches of thirteen populations of brook trout: prey taxonomic composition and prey size distribution. For both taxonomic and size diversity of fish diets, population niche width was positively related to both the within- and between-individual components. For taxonomic diversity, the two components increased in parallel, while for size diversity, the between-individual component became more important relative to the within-individual component in populations with the greatest niche widths. Our results support the Niche Variation Hypothesis that populations with broader niches are more heterogeneous among individuals and show that individual niche width and individual specialization can operate in parallel to expand the population niche.

**Supplementary Information:**

The online version contains supplementary material available at 10.1007/s00442-022-05201-z.

## Introduction

Niche theory has long been a cornerstone of ecological and evolutionary research, and continues to feature prominently in contemporary theory on coexistence, population persistence, and speciation (e.g. Chesson 2000; Chase and Leibold 2003; Ackerman and Doebeli 2004). Studies of the niche traditionally assumed that individuals comprising a population were ecologically identical, but empirical studies have shown that this assumption is often violated in nature (Bolnick et al. 2003). Many generalist populations consist of specialized individuals using distinct subsets of the available resources (Bolnick et al. 2003; Araújo et al. 2011). This niche partitioning among individuals (i.e. “individual specialization”) can comprise considerable proportions of the population niche width, in some cases exceeding the contribution of individual niche width (Bolnick et al. 2003; Araújo et al. 2011).

Individual specialization and diet breadth can have distinct impacts on population dynamics and stability, ecological interactions, and evolutionary transitions. Among-individual variation dampens population fluctuations in response to environmental change (Forsman and Wennersten 2016), stabilizes predator–prey interactions (Doebeli 1997), and also mediates species coexistence and community dynamics (Bolnick et al. 2011). For example, models of predator–prey interactions show that the incorporation of individual specialist predators flips the outcome of indirect interactions between two prey species from mutually antagonistic (i.e. apparent competition) to neutral or facilitative when compared to systems with generalist individuals (Schreiber et al. 2011).

Populations with specialist individuals may also follow distinct evolutionary trajectories from ones composed of generalists. Among-individual niche variation promotes frequency-dependent disruptive selection (Bolnick 2004; Bürger et al. 2006), and can drive the evolution of resource-use polymorphisms or ecological speciation if niche variation is heritable (Rundle and Nosil 2005; Yoder et al. 2010). Individual specialization is also hypothesized to be an adaptive mechanism maintaining standing genetic variation (Van Valen 1965), and may therefore seed processes of evolutionary diversification including adaptive radiations. Identifying the factors affecting the balance of individual specialization vs. individual niche width is therefore critical to a predictive understanding of ecological and evolutionary processes (Bolnick et al. 2003).

Ecological opportunity (i.e. the diversity of available resources) is one factor thought to promote individual specialization. Van Valen (1965) proposed the Niche Variation Hypothesis (“NVH”) that populations with broader niche widths are composed of more specialized individuals after observing that island populations of birds had broader niches than their mainland counterparts and were also more variable morphologically. Van Valen reasoned that when mainland birds colonized island habitats that lacked competitors (i.e. “ecological release”), individual specialization allowed them to expand their population niche to take advantage of unoccupied niche space. Subsequent tests of this hypothesis often failed to find correlations between population niche width and morphological variance (e.g. Soule and Stewart 1970; Meiri et al. 2005). However, morphology may be a poor proxy for resource-use specialization, and tests of the NVH using actual diet measures have sometimes (e.g. Bolnick et al. 2007; Costa et al. 2008; Maldonado et al. 2017) but not always (Diaz 1994; Costa-Pereira et al. 2019) found support. Part of this discrepancy might be attributable to the niche axes measured: The resource-use niche is many dimensional and individuals may partition niche space along any axis, yet most studies measure only a single component of the diet (Ingram et al. 2018).

Besides artifacts of study design, variation in processes of niche formation can explain the mixed empirical support for the NVH. Population niche width (“PNW”) is determined by the combined contributions of niche partitioning among individuals (between-individual component; “BIC”) and individual niche width (within-individual component; “WIC”) (i.e. PNW = WIC + BIC) (Roughgarden 1972). Even in the absence of niche partitioning between individuals, population niche expansion can proceed by the parallel expansion of all individual niches in the population (i.e. “parallel release”). Which of these processes prevails in nature remains an outstanding question.

Theory predicts that all individuals will use the full suite of available resources unless tradeoffs for acquiring alternate resource types make generalization costly (Taper and Chase 1985; Sjödin et al. 2018). Given such tradeoffs, niche expansion following ecological release is predicted to proceed strictly via parallel release (i.e. increased WIC without a concomitant increase in BIC) up to some critical level of WIC determined by the taxa-specific cost of generalization (Sjödin et al. 2018). Once that threshold is reached, niche expansion proceeds strictly by individual specialization (increased BIC without increased WIC) (Sjödin et al. 2018). While some authors have challenged the notion that increases in WIC and BIC do not occur in parallel (e.g. Costa-Pereira et al. 2019; Herrmann et al. 2020), a meta-analysis with data from fifteen taxonomic groups found no evidence for a correlation between the two niche components (Sjödin et al. 2018), suggesting that aspects of populations or the environment that promote BIC or WIC (and therefore PNW) tend to vary independently of one another.

Here, we tested the relative contributions of individual niche width (WIC) and niche partitioning among individuals (BIC) across a natural gradient of population niche width in brook trout (*Salvelinus fontinalis*) introduced to previously fishless lakes in California’s Sierra Nevada. We compared the diet composition of 229 individual brook trout from thirteen lakes spanning an 830 m elevation gradient. In each lake, we quantified PNW, WIC, and BIC for two axes of the brook trout niche: prey taxonomic composition and prey size distribution. (Note that size distribution is a univariate axis while prey composition is a multivariate description of the taxonomy of the prey that make up the diet. We refer to both as “niche axes” for simplicity.) We also measured PNW based on stable carbon and nitrogen isotope ratios to evaluate the relationship between long- and short-term measures of resource use. We characterized variation in PNW with elevation and lake size. We then evaluated how the proportional contributions of WIC and BIC covaried among lakes and the contribution of each to PNW. This allowed us to test both the Niche Variation Hypothesis (Van Valen 1965) that individual specialization increases with PNW, as well as the prediction from theoretical models that BIC & WIC are independent across populations (Sjödin et al. 2018). Finally, we evaluated the relationship between niche width and niche position to ask how diet composition and diversity covary.

## Methods

### Natural history

On the eastern slope of California’s Sierra Nevada mountains, high elevation post-glacial lakes were historically fishless. Many of these lakes were stocked with fish beginning in the late 1800s to support recreational fisheries. In Yosemite National Park and some surrounding areas, fish stocking ceased for most water bodies in the 1970s (and for all by 1991), but many naturally reproducing brook trout populations persist (Knapp 1996). The elevation gradient in this region generates variation in abiotic conditions and community composition, and therefore ecological opportunity for potential colonists. This ecological variation, paired with the recent and largely synchronous colonization of these lakes by brook trout of hatchery origin, provides a natural gradient in which to test questions related to variation in population niche width.

### Sample collection and laboratory processing

We collected 229 brook trout from thirteen lakes in and around Yosemite National Park, California, USA from July–August, 2018 by angling (Fig A1, Appendix 1 in Supplementary Information). Lakes ranged in elevation from 2508–3337 m and in surface area from 2 to 21 ha (see Table A1 for details on individual lakes). One to three anglers circled the lake from shore, using similar flies/lures at each site. We used an inflatable raft to sample any areas that could not be effectively sampled from shore. We immediately euthanized fish in the field via blunt force trauma to the head followed by manual pithing in accordance with UCSD IACUC protocol #S14140 and froze them at − 20 °C upon return to the field station. We later processed each fish by thawing, weighing to the nearest centigram (wet weight), measuring to the nearest millimeter (standard length), and dissecting to extract stomach contents, which we preserved in 70% ethanol. We collected muscle tissue samples for stable isotope analysis (to relate short- and long-term diets; see below) from each fish below the dorsal fin but above the lateral line. We dried the tissue samples at 60 °C, ground them to a fine powder, and sent them to the Stable Isotope Facility at the University of California, Davis for analysis of δ^13^C and δ^15^N.

To characterize fish diets, we identified individual prey items from stomach contents to order, life stage (juvenile or adult), and habitat (terrestrial or aquatic) and enumerated them based on whole individuals or reconstructions from identifiable parts. We photographed prey items under a microscope and measured them digitally along their longest axis. We did not measure prey items that were too digested to identify or measure. Fish with empty stomachs were excluded from the analysis.

### Characterizing the population niche and its components

We evaluated two axes of the brook trout trophic niche: prey taxonomic composition and prey size structure. For both niche axes, measures of the total population niche width (PNW) can be decomposed into two components: the within-individual component (WIC) and the between-individual component (BIC), where WIC + BIC = PNW (Roughgarden 1972). Greater BIC indicates more individual specialization, while greater WIC indicates greater individual niche width. We evaluated the relative importance of these two components using the proportion of PNW attributable to within- and between-individual components (WIC/PNW and BIC/PNW, respectively). We use subscripts to denote the niche axis to which each metric refers (e.g. PNW_size_ and PNW_taxa_ refer to the population niche width of the prey size axis and the prey taxa axis, respectively).

We used the R package ‘RInSp’ v1.2.4 to calculate PNW, WIC, and BIC (Zacarelli et al. 2013). For the prey size axis of the brook trout niche, we calculated PNW_size_—the variance in prey size for every measurable prey item found in the stomach contents of every individual sampled from the population—and its decomposition into WIC_taxa_ and BIC_taxa_ (Roughgarden 1972)—using the ‘WTcMC’ function in ‘RInSp’, with individuals weighted by number of prey items consumed (Zacarelli et al. 2013). We calculated PNW_taxa_—the Shannon–Weaver diversity index—as well its decomposition into WIC_size_ and BIC_size_ (Roughgarden 1979; Bolnick et al. 2002)—using the ‘WTdMC’ function in ‘RInSp’ (Zacarelli et al. 2013). (Note that our measures of PNW_taxa_ and PNW_size_ are not directly comparable as the latter uses a single continuous variable that can be represented by the dispersion of the distribution in contrast to the compositional diet data.) We also used these functions to test the null hypothesis of no individual specialization in each population, for each niche axis. These tests compare the observed values of WIC_size_ and WIC_taxa_ to null distributions constructed with Monte Carlo resampling procedures in which generalist consumers sample randomly from a shared prey distribution (Zacarelli et al. 2013). We calculated null BIC values for each population as the mean of the resampling distribution and plotted these values alongside observed values to facilitate evaluation of sampling effects.

For the taxonomic niche axis, measures of BIC_taxa_ may be inflated by consumers that use only a single resource (Bolnick et al. 2002; Zacarelli et al. 2013). For this reason, we calculated a second measure of individual specialization— an adjusted version of Araújo’s E—developed specifically for evaluating the degree of individual specialization in categorical datasets (Araújo et al. 2008). E represents the mean among-individual diet dissimilarity and has potential values ranging from 0 to 1, where 1 indicates complete specialization and 0 indicates identical diets for every individual in the population (Araújo et al. 2008). We used an adjusted version of the metric (*E*_adj_) that rescales *E* based on a null value *E*_null_ calculated by a Monte Carlo resampling procedure that assumes every individual prey capture was randomly sampled from a shared distribution (Zacarelli et al. 2013). The resulting *E*_adj_ ranges from 0 when *E* = *E*_null_ to 1 when individual diets have no overlap (Zacarelli et al. 2013). The adjustment allows us to compare across lakes over which *E*_null_ varies. *E*_adj_ was calculated using ‘RInSp’ v1.2.4 (Zacarelli et al*.* 2013). We use *E*_adj_ rather than BIC/PNW_taxa_ for significance tests of individual specialization for the taxonomic niche axis.

To test the relationship between short-term and longer-term diets, we compared our cross-sectional data from stomach contents to individual stable C and N isotope ratios which provide an integrative measure of diet over weeks to months (Peterson and Fry 1987; Bolnick et al. 2002), with in situ measures of muscle tissue isotopic turnover exceeding 5 months in coldwater rainbow trout (Skinner et al. 2017). We tested for a relationship between diet and muscle tissue stable isotope ratios using permutational analysis of variance (PERMANOVA) with diet composition as the multivariate response, and δ^13^C and δ^15^N values as explanatory variables using the ‘vegan’ package v2.5–6 in R (Oksanen et al. 2019). We also measured PNW in isotopic space (PNW_iso_) as the area of ellipses that captured 95% of the population variance in bivariate isotope space (δ^13^C and δ^15^N) (Jackson et al. 2011). Ellipses were fit to the population isotope data as bivariate normal distributions using Bayesian estimation with vague priors; fitting was conducted using the R package ‘SIBER’ v2.1.4 (Jackson et al. 2011). The use of Bayesian inference to estimate ellipse area allows for robust comparison across datasets with varying sample size (Jackson et al. 2011). We used pairwise comparisons of PNW_iso_, PNW_taxa_, and PNW_size_ to evaluate complementarity of information contained in each axis.

To test the effects of lake elevation and surface area on brook trout PNW, we used multiple linear regression with standardized predictors, run separately for PNW_size_ and PNW_taxa_. 

### Testing the Niche Variation Hypothesis

Van Valen’s (1965) Niche Variation Hypothesis predicts a positive relationship between PNW and individual specialization. We tested this prediction for both the prey size distribution and the prey taxonomic composition axes of the brook trout niche using linear regression of BIC vs. PNW, where a significantly positive relationship (with α = 0.05) was taken as support for the hypothesis. We also tested the relationship of BIC/PNW—which some studies use as the metric for individual specialization—vs. PNW.

Some of our response variables of interest (BIC/PNW_size_, BIC/PNW_taxa_, and E_adj_) assume continuous values in the open interval (0,1). Accordingly, we modeled these relationships using beta regression which assumes a beta-distributed response variable with a mean that’s related to linear predictors through a link function (similar to generalized linear models) (Cribari-Neto and Zeileis 2010). Beta regression reduces bias compared to modeling transformed response variables (Douma and Weedon 2019). We fit beta regression models using the ‘betareg’ package in R (Cribari-Neto and Zeileis 2010). We fit models with several different link functions and used Akaike’s Information Criterion (AIC) to identify the best fit models (Douma and Weedon 2019).

### Testing the independence of individual specialization and individual niche expansion

Sjödin et al. (2018) hypothesize on theoretical and empirical grounds that niche expansion arises via either individual specialization (BIC) or individual niche expansion (WIC), but not both in parallel. This leads to the testable prediction that WIC and BIC are uncorrelated (Sjödin et al. 2018). For both the size and taxonomic axes of the brook trout niche, we tested the null hypothesis that the true correlation is equal to zero using Pearson’s product moment correlation with α = 0.05.

### Evaluating change in niche position during niche expansion

To evaluate whether population niche position shifts during niche expansion, we tested the relationship between population niche width (PNW_size_ or PNW_taxa_ for size and taxonomic dimensions, respectively) and niche position for both niche axes (prey size distribution and prey taxonomic composition). For prey taxonomic composition, niche position was determined by ordination of the brook trout diet matrix using non-metric multidimensional scaling (NMDS). We used a Bray–Curtis dissimilarity matrix and calculated three NMDS dimensions (*k* = 3). The algorithm found a convergent solution with stress < 0.15. The final configuration was rotated such that the variance of points was maximized along NMDS1. We conducted the ordination using the ‘metaMDS’ function with the R package ‘vegan’ v2.5–6 (Oksanen et al. 2019).

## Results

### Variation in population niche width

**Fig. 1:**
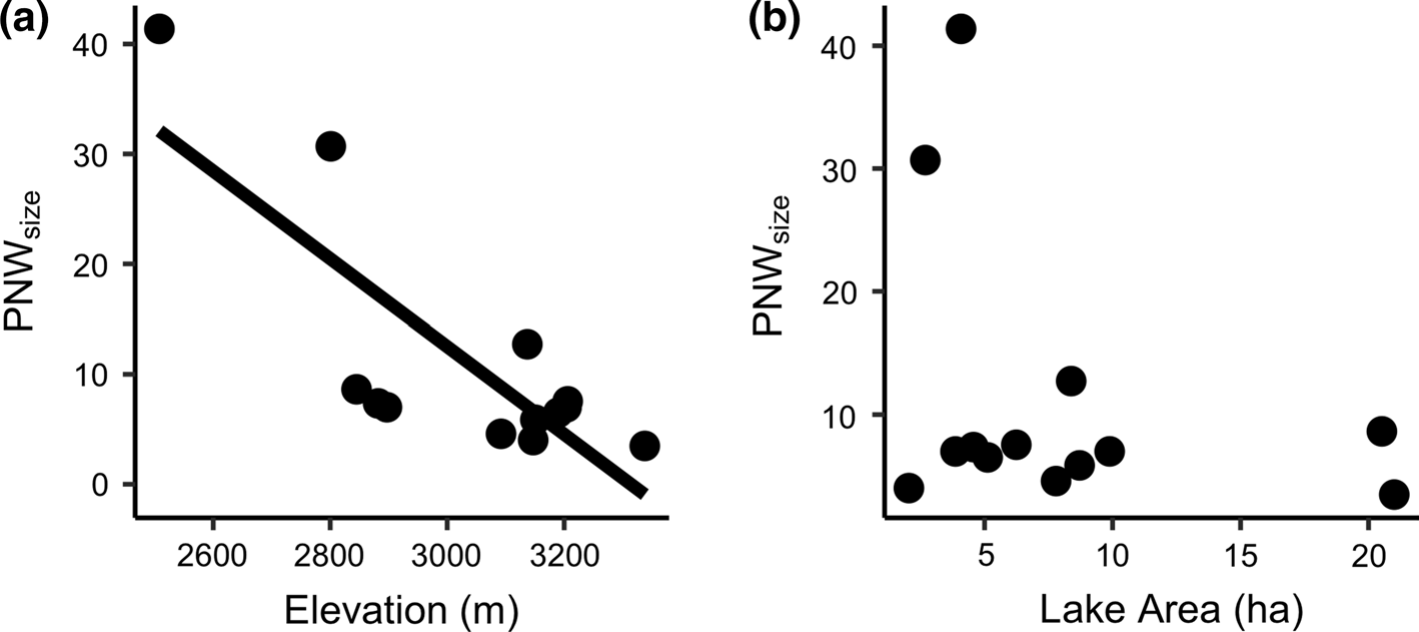
Variation in population niche width with **a** lake elevation and **b** lake area for the prey size axis of the brook trout resource-use niche (*N* = 13). Relationships with PNW_taxa_ and PNW_iso_ were not significant (see Fig. A2 in Appendix S1)

Population niche width was highly variable across lakes for both niche axes: PNW_size_ varied by a factor of 12 across lakes (range: 3.48–41.37) and PNW_taxa_ varied by a factor of 8 (range: 0.25–1.98) (Table A1; see Figs. A3 and A4 for individual diet compositions). PNW_size_ decreased with elevation (*P* < 0.01) but was not affected by lake area (Table 1; Fig. 1A–B). Variation in PNW_taxa_ and PNW_iso_ was not explained by elevation or lake area (Table 1; Fig. A2C–F). PERMANOVA showed a significant relationship between isotopic predictors (δ^15^N and δ^13^C) and diet taxonomic composition, indicating a relationship between long and short-term diet measures (δ^13^C: F_1,222_ = 5.61, *P* < 0.001; δ^15^N: F_1,222_ = 9.03; *P* < 0.001), but note that unmeasured differences in isotopic baselines may contribute to this effect.

**Table 1:** Regression models for variables of interest. Correlation results are only reported in the main text

Figs.	Response variable	Model type (link)	Predictor variable	Effect size	Effect SE	*t* or *z* value	*P*	*F* (df)	R^2^*
1A	PNW_size_	Linear (identity)	**Elevation**	**− 8.70**	**2.14**	**− 4.1**	** < 0.01**	9.81 (2, 10)	0.66
			Lake Area	**− **2.06	2.14	**− **0.96	0.36		
1B	PNW_taxa_	Linear (identity)	Elevation	0.43	1.05	0.41	0.69	0.20 (2, 10)	0.04
			Lake Area	0.42	1.05	0.40	0.70		
1C	PNW_iso_	Linear (identity)	Elevation	0.15	0.13	1.18	0.27	1.41 (2, 10)	0.22
			Lake Area	0.12	0.13	0.95	0.36		
2A	BIC_size_	Linear (identity)	**PNW**_**size**_	**0.73**	**0.03**	**26.94**	** < 1 × 10**^**–10**^	725.80 (1, 11)	0.99
2A	WIC_size_	Linear (identity)	**PNW**_**size**_	**0.27**	**0.03**	**9.77**	** < 1 × 10**^**–6**^	95.5 (1, 11)	0.90
2D	BIC_taxa_	Linear (identity)	**PNW**_**taxa**_	**0.45**	**0.08**	**6.03**	** < 0.0001**	36.30 (1, 11)	0.77
2D	WIC_taxa_	Linear (identity)	**PNW**_**taxa**_	**0.56**	**0.08**	**7.28**	** < 0.0001**	52.94 (1, 11)	0.83
2B	BIC/ PNW_size_	Beta (loglog)	**PNW**_**size**_	**0.38**	**0.14**	**2.76**	** < 0.01**	NA	0.52
2E	BIC/ PNW_taxa_	Beta (logit)	PNW_taxa_	0.03	0.19	0.16	0.87	NA	0.00
N/A	E_adj_	Beta (logit)	**PNW**_**taxa**_	**0.53**	**0.24**	**2.17**	** < 0.05**	NA	0.32
3A	Median prey length	Linear (identity)	**PNW**_**size**_	**0.20**	**0.04**	**4.85**	** < 0.001**	23.56 (1, 11)	0.68
3B	NMDS1	Linear (identity)	**PNW**_**taxa**_	**− 0.52**	**0.11**	**− 4.58**	** < 0.001**	20.98 (1, 11)	0.66

Table shows the figure showing the corresponding data, the response (dependent) and predictor (independent) variables, the link function used in the model, the effect size (slope estimate), error, *t* or *z* value (for linear and beta regression models, respectively), the *p* value for that predictor’s effect, the *F* statistic and degrees of freedom (numerator, denominator) for the complete model (for linear models), and the *R*^2^ or pseudo-*R*^2^ value (for linear and beta regressions, respectively). Significant predictors (α = 0.05) are bolded

*Pseudo-R^2^ values are reported for beta regression models

### Mechanisms of population niche expansion

**Fig. 2:**
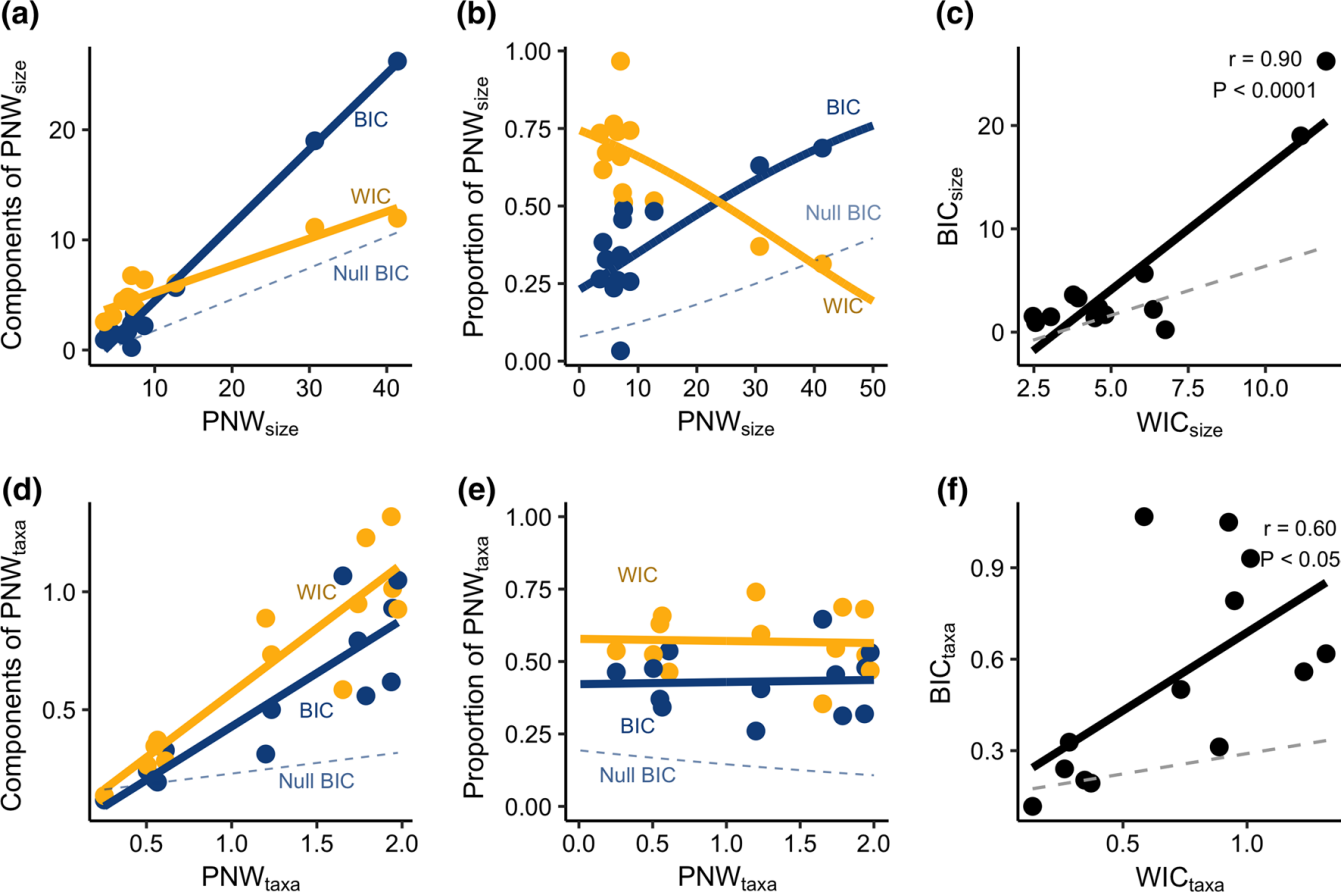
Mechanisms underlying brook trout population niche expansion for two niche axes: prey size distribution (**a**–**c**) and prey taxonomic composition (**d**–**f**) (*N* = 13). Left panels (**a**, **d**) show absolute change in the between- and within-individual niche components (BIC and WIC, respectively) during population niche expansion. Middle panels (**b**, **e**) show the proportional contribution of each component during niche expansion. Dashed lines show null expectations for BIC, demonstrating that specialization is greater than expected due to sampling effects alone. Right hand panels show the relationship between WIC and BIC (solid line), testing the theoretical prediction of no correlation, as well as the relationship between WIC and the null expectation for BIC (dashed line). Note that PNW_size_ and PNW_taxa_ are not directly comparable because they use different data types and metrics of niche width

Niche partitioning between individuals (BIC) and individual niche width (WIC) both increased with population niche width for both the prey taxonomic composition and prey size axes of the brook trout niche (Fig. 2A, D; Table 1). With broader population niches, BIC became increasingly more important than WIC for prey size (Beta regression; *p* < 0.01) (Fig. 2B; Table 1), but the proportional contributions of the two components were similar and invariant with population niche width for prey taxonomic composition (Fig. 2E; Table 1). Our other metric of individual specialization on prey taxa E_adj_ was positively related to PNW_taxa_ (Beta regression *P* < 0 0.05; Table 1), but note that this metric does not reflect the proportional contribution of individual specialization to population niche width. Thus, our results were consistent with the Niche Variation Hypothesis for prey size but not prey taxa. Across both the size and taxonomic axes, the observed trends in BIC were distinct from those fitted to the means of null distributions calculated from Monte Carlo resampling of prey items from each population (dashed lines in Fig. 2), indicating that our results are not attributable to sampling effects.

Relative to null distributions that assumed individuals sampled randomly from a shared distribution of prey items, brook trout showed significant individual specialization on prey size (BIC/PNW_size_) in 69% of lakes, and on prey taxa (*E*_adj_) in 31% of lakes (α = 0.05) (Table A1) (Note that *E*_adj_ was used for the significance test because it reduces bias of monophagous individuals relative to BIC/PNW_taxa_; see “Methods”). The degree of individual specialization varied across lakes for both prey taxa and prey size axes of the brook trout niche: For prey taxa, the mean adjusted pairwise dissimilarity in diet ranged from 0.01 to 0.77 (mean ± 1 SD: *E*_*ad*j_ = 0.42 ± 0.21), and between-individual differences accounted for 26–65% of PNW_taxa_ (mean ± 1 SD: BIC/PNW_taxa_ = 0.43 ± 0.11); for prey size, between-individual differences in diet accounted for 3–69% of total population variance (mean ± 1 SD: BIC/PNW_size_ = 0.37 ± 0.18) (Table A1).

In contrast to the theoretical prediction from Sjödin et al. (2018), WIC and BIC were positively correlated for both the prey size (*r* = 0.90; *P* < 0.0001) and prey taxa (*r* = 0.60; *P* < 0.05) axes of the brook trout niche (Fig. 2C, F).

### Shifts in niche position with niche expansion

**Fig. 3:**
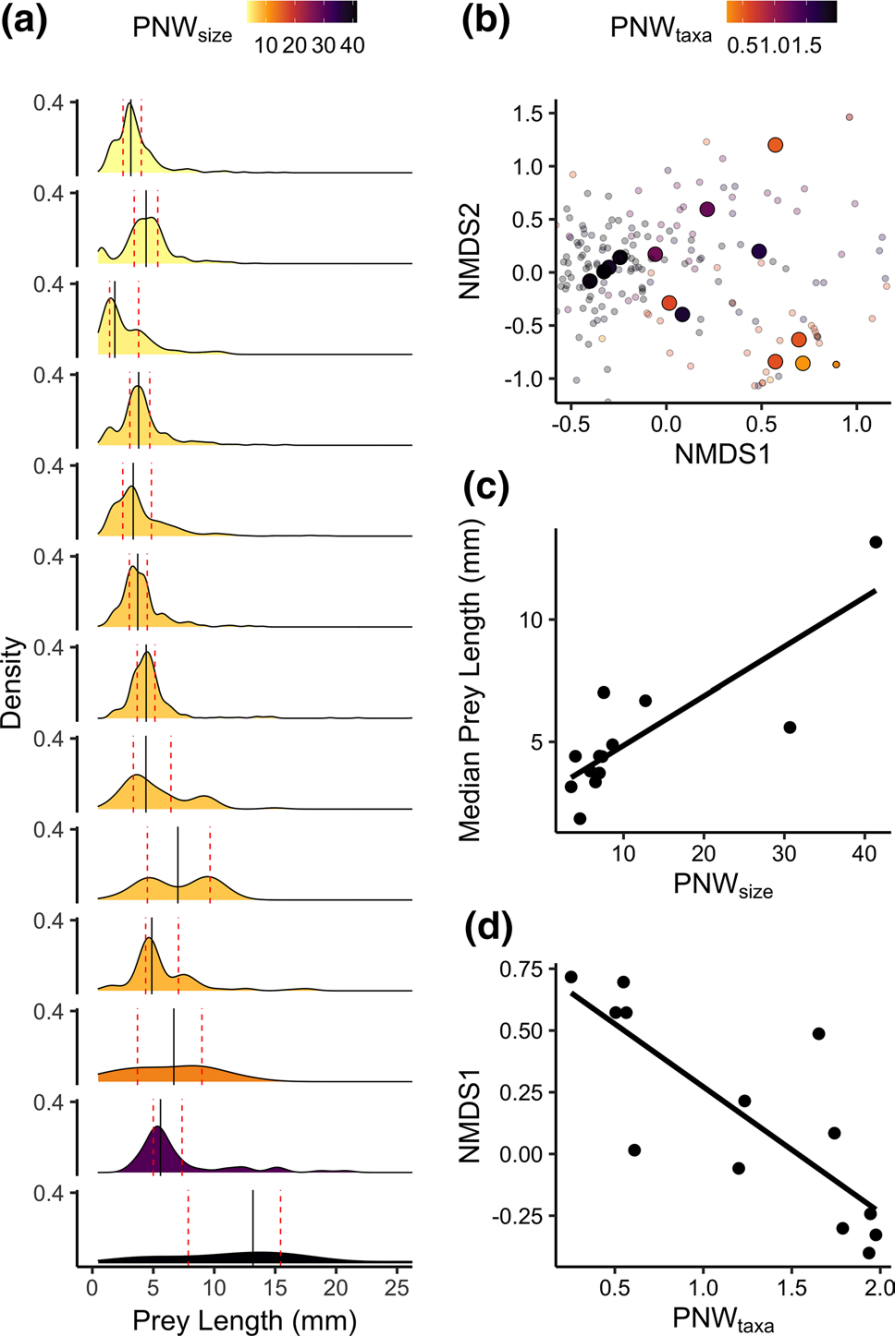
Shifts in brook trout niche position with niche expansion for **a**, **c** the prey size niche axis and **b**, **d** prey taxonomic niche axis. **a** Density plots of prey length for each of the 13 populations, arranged in increasing order of PNW_size_ moving from top to bottom. Solid vertical lines show the median length, dashed red lines show the 1st and 3rd quartiles. **b** Ordination plot of brook trout diet taxonomic composition from non-metric multidimensional scaling. Small points show individual fish (*N* = 229), large points show population mean values colored by PNW_taxa_ (*N* = 13). **c** Median prey length increases linearly with increasing variance in prey length (population niche width; PNW_size_) (*N* = 13). **d** Taxonomic niche position, determined by the mean position along the primary axis of variation from the ordination (NMDS1), shifts linearly with increasing Shannon–Weaver diversity index (population niche width; PNW_taxa_) (*N* = 13)

Average population niche position changed with niche expansion for both the prey size and prey taxa axes of the brook trout niche. Median prey length increased linearly with variance in prey length (i.e. PNW_size_) (Fig. 3A, C; Table 1). Niche position for the taxonomic axis was determined as the position along the primary axis of variation from an ordination of the brook trout diet matrix (see “Methods”) (Fig. 3B). NMDS1 was negatively related to terrestrial insects, annelids, bivalves, and aquatic beetles and was positively related to odonate larvae, with smaller aquatic insect larvae occupying intermediate positions (e.g. diptera, ephemeroptera). Position along this axis decreased linearly with increasing PNW_taxa_ (Fig. 3D; Table 1).

## Discussion

The extent to which population niche width is driven by niche partitioning between individuals versus individual niche width affects ecological outcomes and evolutionary trajectories (Bolnick et al. 2011; Araújo et al. 2011). The Niche Variation Hypothesis predicts that the between-individual component should increase in systems with broader population niches, but broader individual niches can also support broader population niches. We characterized the relative contribution of within- and between-individual niche components for two niche axes in a comparative study of thirteen populations of brook trout introduced to previously fishless mountain lakes. We showed that populations with broader niches were composed of more generalist individuals, and also individuals that were more differentiated in use of different prey. This suggests that niche width in brook trout is driven by both individual niche expansion and niche partitioning among individuals, and that these mechanisms operate in parallel.

### Environmental effects on population niche width

Population niche width was highly variable across lakes for both niche axes, providing a robust gradient for our comparative study. We found that environmental characteristics predicted population niche width for prey size but not prey taxa, and that the widths of these two niche axes were uncorrelated. The population niche for prey size became narrower with increasing elevation, and prey became smaller on average, while the width of the taxonomic niche axis showed no relationship. We observed a shift in niche position with population niche expansion for both axes, where populations with broader niches had larger median prey sizes and also occupied distinct positions in ordinated taxonomic niche space. While some larger prey taxa are more common at lower elevation sites (e.g. odonates), much of the increased variation in prey size occurs within taxonomic groups as well. High elevation habitats pose a number of challenges for their inhabitants including high ultraviolet radiation, low temperatures, and short growing seasons that may act as abiotic filters on prey traits. These stressors have been shown to reduce functional or taxonomic diversity in a number of systems including terrestrial plants (Read et al. 2014), ants (Reymond et al. 2013), lake bacterioplankton (Li et al. 2017), and lake zooplankton (Hessen et al. 2006), among others, and appear to reduce size (but not taxonomic) diversity in macroinvertebrate prey in our system (including aquatic and terrestrial insects). This interpretation assumes a positive coupling between the diversity of consumed prey and diversity of the invertebrate assemblage, which remains untested as we did not measure the latter. It is also possible that the differences in the size diversity of invertebrate prey that we observed across the elevational gradient were driven by predator preferences rather than invertebrate diversity. Nonetheless, our interpretation is broadly consistent with prior studies that have shown that niche width and ecological opportunity decline at higher latitudes (Araújo and Costa-Pereira 2013; Yurkowski et al. 2016), as temperature change is a dominant feature of both latitudinal and elevational gradients. Additional work is required to assess whether temperature control of resource functional diversity is a general phenomenon.

Surprisingly, we found no effect of lake size (surface area) on population niche width. These results contrast with Bolnick and Ballare (2020) who showed that ecological opportunity and individual specialization of stickleback varied with lake area, peaking in intermediate sized lakes. They attributed this quadratic relationship to the more even balance of benthic and limnetic habitat and therefore prey available in intermediate sized lakes. Mountain lakes like the ones we studied tend to be quite small (< 30 ha), shallow, and oligotrophic (Piovia-Scott et al. 2016). These lakes occupy the very left (small) side of the lake surface area distribution evaluated in Bolnick and Ballare (2020). Further, the relative availability of limnetic and benthic habitat may scale differently with lake size in our system, since the photic zone extends to the deepest points of many of our lakes. Lastly, unlike stickleback, the brook trout in our study are largely insectivorous, and do not rely on zooplankton prey as adults, the predominant limnetic resource. Thus, our results do not conflict conceptually with prior studies, but show that empirical studies are needed to build a more general understanding of environmental control of population niche width across a range of systems with varying natural histories (Araújo et al. 2011; Ingram et al. 2018).

### Drivers of population niche width

Recent theoretical work suggests that niche expansion proceeds by two orthogonal processes: strictly individual niche expansion (i.e. parallel release) or strictly niche partitioning between individuals (Sjödin et al. 2018). Their models show that with increasing resource diversity, individual niches should expand (without niche partitioning among individuals) until some critical threshold, beyond which generalization becomes costly and niche expansion occurs strictly by niche partitioning among individuals. Evolutionarily stable strategies in terms of relative values of WIC and BIC depend on the shape of the tradeoff function describing the cost of generalization, and the resource diversity available in the environment. They show a transition in the values of these two parameters where niche expansion occurs only through evolution of WIC or BIC but not both. Their work is consistent with prior models indicating that niche partitioning only occurs when there is a cost to individual generalization (Ackerman and Doebeli 2004), but the implication that, given such costs, these processes occur orthogonally rather than in parallel is novel. In our populations, resource diversity in the environment is likely to be the major factor determining PNW since the same constraints on specialization and generalization likely apply to all brook trout populations, but these populations vary in terms of the resource diversity available in their environment.

This prediction has been tacitly challenged by some authors (e.g. Costa-Pereira et al. 2019; Herrmann et al. 2020), but a meta-analysis by Sjödin et al. (2018) of fifteen taxonomic groups for which suitable data were available showed that most species tend to vary in either BIC or WIC but not both, and the two were uncorrelated, in agreement with their theoretical predictions. Here, we provided one counter example where the within- and between-individual components of the total population niche width are correlated across a wide range of population niche widths from thirteen populations, for two niche axes. In addition, the two components contributed similarly to PNW, although for size diversity, the contribution of WIC declined at the highest levels of PNW, perhaps reflecting stronger constraints on prey with large variation in size relative to variation in taxonomic composition. Our results suggest that the niche evolution model employed by Sjödin et al. (2018) may apply to many species but is not universally generalizable, and that niche expansion can proceed by a combination of individual niche expansion and partitioning of niche among individuals in some systems, including ours.

### Relation to the Niche Variation Hypothesis

Prior empirical tests of the Niche Variation Hypothesis that populations become more heterogeneous as they expand their niches in response to ecological opportunity (Van Valen 1965) have found both opposing (e.g. Soule and Stewart 1970; Diaz 1994; Meiri et al. 2005) and supporting evidence (e.g. Bolnick et al. 2007; Costa et al. 2008; Hsu et al. 2014; Maldonado et al. 2017). Our results add to the body of supporting evidence. We found that niche partitioning between-individual brook trout is stronger in populations with broader niches. For the prey size axis of the brook trout niche, this between-individual partitioning comprised an increasingly greater proportion of the population niche width as population niche width increased. For the prey taxa niche axis, proportional contributions of the within-individual niche expansion and between-individual niche partitioning were invariant with population niche width.

## Conclusions

By showing that the within- and between-individual niche components are correlated across a gradient of population niche width in brook trout introduced to previously fishless lakes, we provide an empirical counterexample to models and prior empirical work indicating that individual niche expansion and individual specialization are mutually exclusive processes. Populations with higher diet diversity in terms of prey size and taxonomic variation tend to contain fish with more variable diets *and* greater fish-to-fish differences in diet composition. The only aspect of the environment to consistently predict niche position and variation was elevation, as fish tend to eat smaller prey that vary less in size in higher elevation lakes. Our results support the Niche Variation Hypothesis, showing that individual specialization increases with population niche width, even as individual niche width also increases.

## Supplementary Information

Below is the link to the electronic supplementary material.

Supplementary file1 (PDF 584 KB)

## Data Availability

All data are available at https://doi.org/10.5061/dryad.wpzgmsbps.

## Code availability

Code is available at https://doi.org/10.5061/dryad.wpzgmsbps.
