# Supplementary material for: Population niche width is driven by within-individual niche expansion and individual specialization in introduced brook trout in mountain lakes
Source: Oecologia. 2022 Jun 3;200(1-2):1–10. doi: 10.1007/s00442-022-05201-z (PMC9547792; doi:10.1007/s00442-022-05201-z)
Supplement: Supplementary file 1 — Supplementary file1 (PDF 584 KB) [file 442_2022_5201_MOESM1_ESM.pdf]

Electronic Supplementary Material for:

Population niche width is driven by within-individual niche expansion and individual specialization in introduced Brook Trout in mountain lakes

Authors:

H. K. Baker[1]* (0000-0002-0166-5085);

C. E. F. Bruggeman[1] (0000-0003-4846-4915);

J. B. Shurin[1] (0000-0001-7870-1972)

[1] Section of Ecology, Behavior, and Evolution, University of California San Diego, California 92092, USA.

*corresponding author: hkbaker@ucsd.edu

Appendix S1

Additional Tables and Figures

Table A1. Environmental characteristics and population niche composition of Brook Trout for prey size and taxonomic diversity. P-values denote the probability that the degree of individual specialization (BIC/PNW_{size} or E_{adj}) is significantly greater than expected if individuals sample randomly from a shared distribution.

**Lake**	**Elevation (m)**	**Surface Area (ha)**	**Sample Size**	**PNW_{size}**	**WIC/PNW_{size}**	**BIC/PNW_{size}**	**P-value [BIC/PNW_{size}]**	**PNW_{taxa}**	**WIC/PNW_{taxa}**	**BIC/PNW_{taxa}**	**E_{adj}**	**P-value [E_{adj}]**
Lukens	2508	4.08	7	41.38	0.69	0.31	0.06	0.50	0.48	0.52	0.78	1.00
Sunrise 1	2801	2.69	18	30.69	0.63	0.37	**<0.05**	1.65	0.65	0.35	0.55	**<0.05**
May	2845	20.52	16	8.64	0.26	0.74	**<0.05**	0.55	0.37	0.63	0.18	1.00
Sunrise 3	2882	4.57	19	7.35	0.46	0.54	0.06	1.23	0.41	0.59	0.45	**<0.05**
Elizabeth	2897	9.89	11	7.00	0.03	0.97	0.16	1.20	0.26	0.74	0.45	1.00
Greenstone	3092	7.79	11	4.59	0.33	0.67	**<0.05**	1.74	0.45	0.55	0.52	**<0.01**
Steelhead	3137	8.38	5	12.73	0.48	0.52	0.16	0.56	0.34	0.66	0.10	0.83
Cascade	3147	2.04	12	4.01	0.38	0.62	**<0.001**	0.61	0.54	0.46	0.57	1.00
Gaylor 1	3150	8.71	42	5.86	0.24	0.76	**<0.001**	1.94	0.48	0.52	0.48	1.00
Spillway	3190	5.12	18	6.51	0.26	0.74	**<0.001**	1.98	0.53	0.47	0.52	**<0.001**
Gaylor 2	3204	3.86	28	7.00	0.34	0.66	**<0.05**	1.79	0.31	0.69	0.35	0.84
Gardisky	3206	6.24	9	7.54	0.49	0.51	**<0.001**	0.25	0.46	0.54	0.01	0.84
Helen	3337	21.00	33	3.48	0.26	0.74	**<0.001**	1.94	0.32	0.68	0.38	0.98

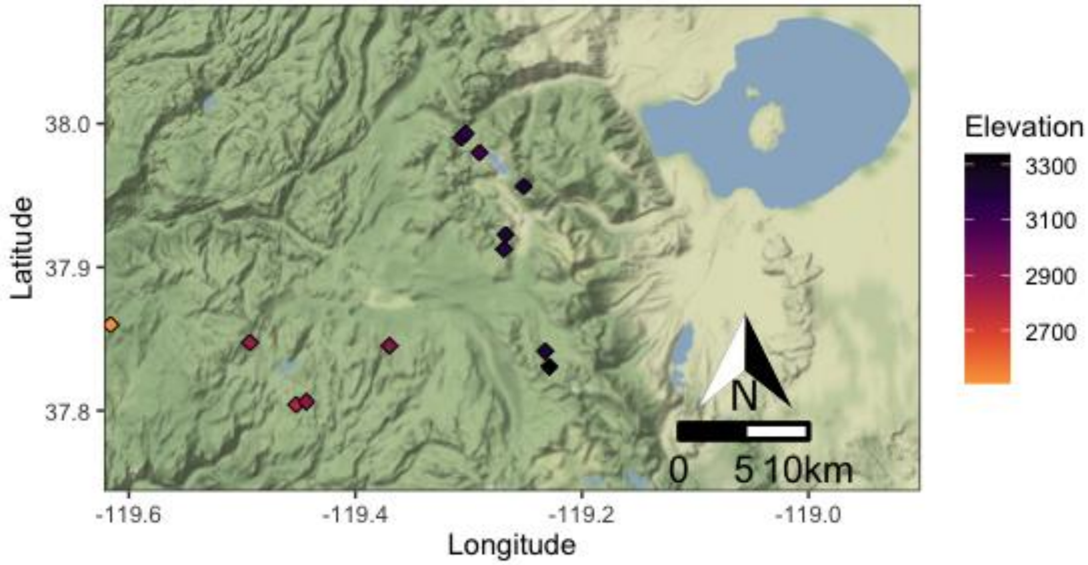

Figure A1. Map of study sites in and near Yosemite National Park, California, USA, with elevation shown in meters.

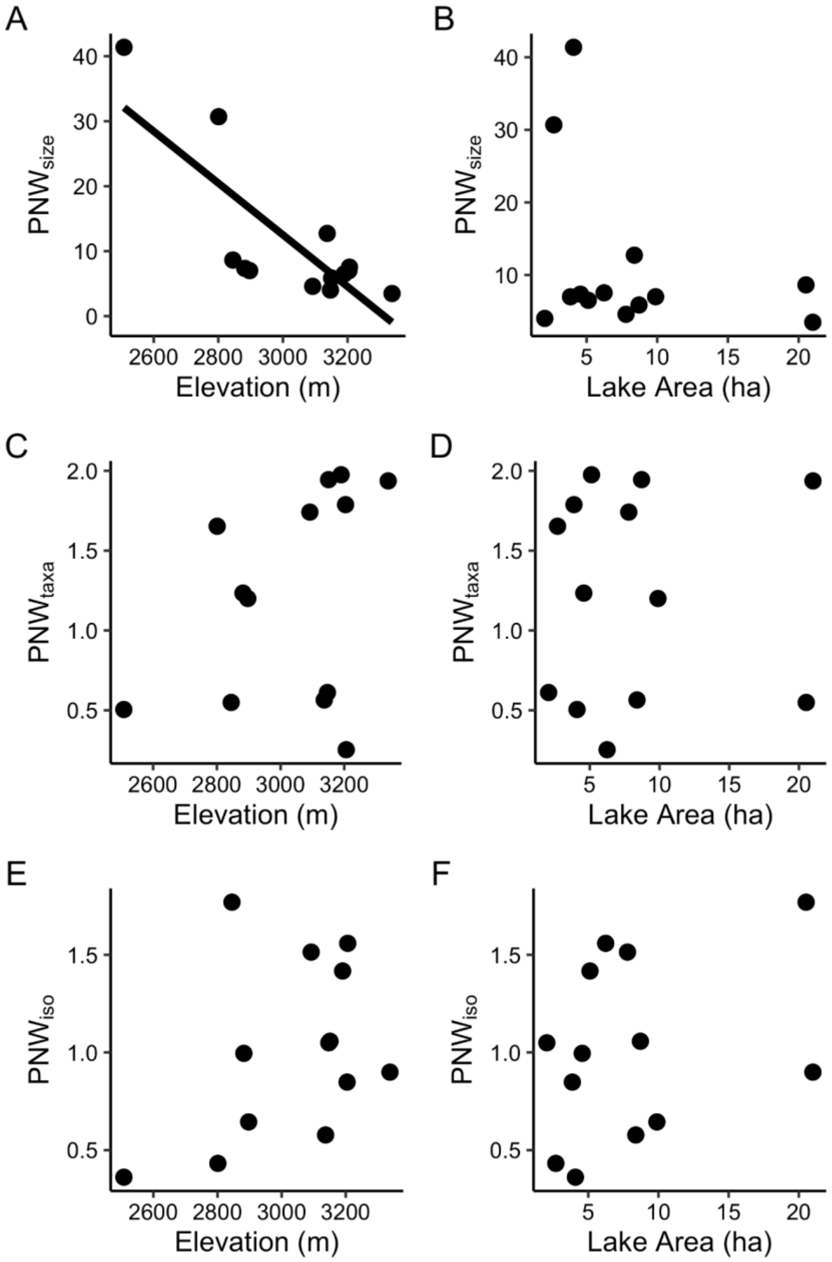

Figure A2. Variation in population niche width with lake elevation and size for three axes of the brook trout resource use niche: prey size distribution (PNW_{size}) (A–B), prey taxonomic composition (PNW_{taxa}) (C–D), and brook trout isotopic composition ($\delta^{13}C$ and $\delta^{15}N$; PNW_{iso}) (E–F). Note that panels A & B are identical to Figure 1 in the main text, but are duplicated here for clarity.

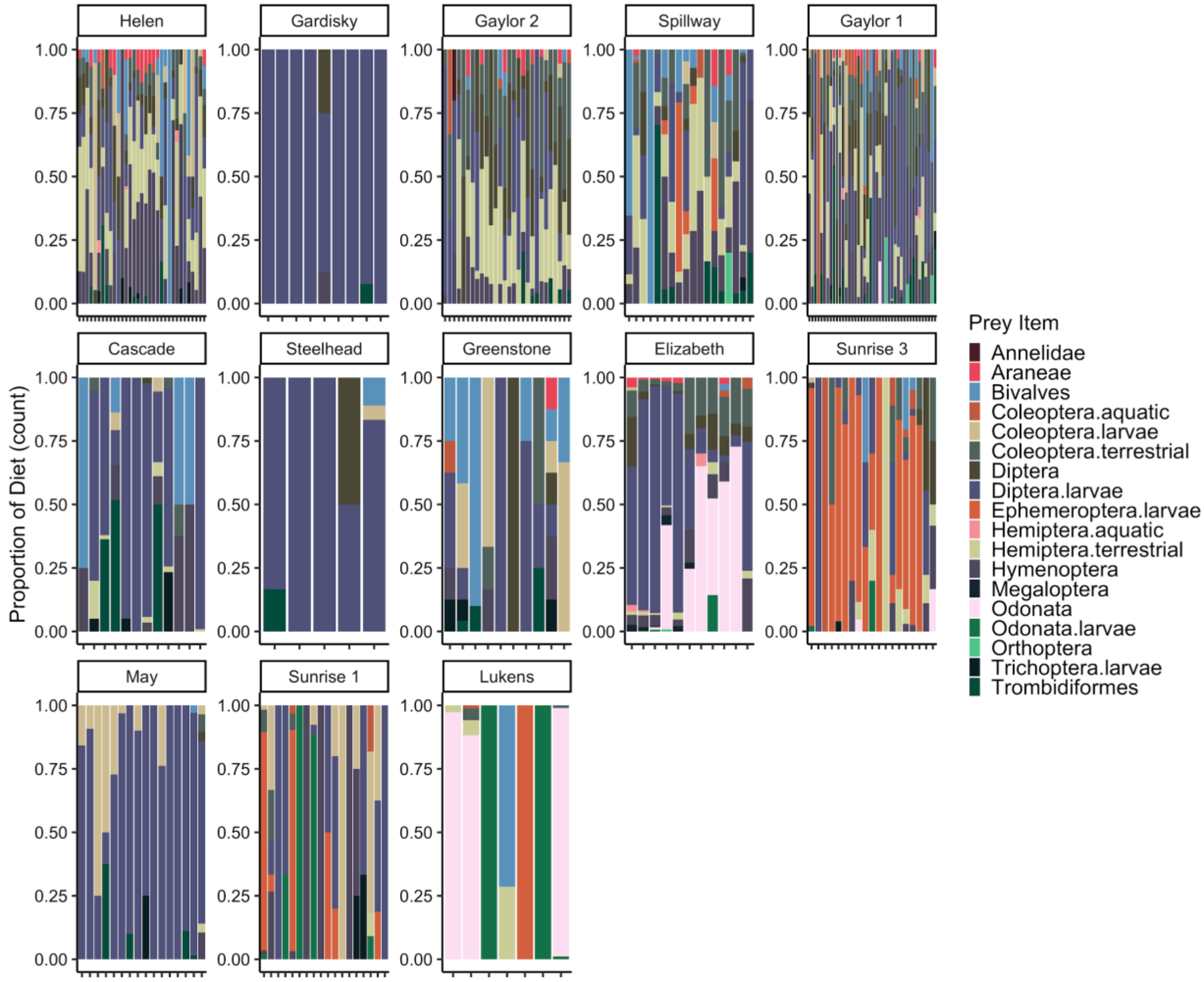

Figure A3. The taxonomic composition of Brook Trout diets for each lake, arranged in descending order of elevation. Each vertical bar represents an individual fish.

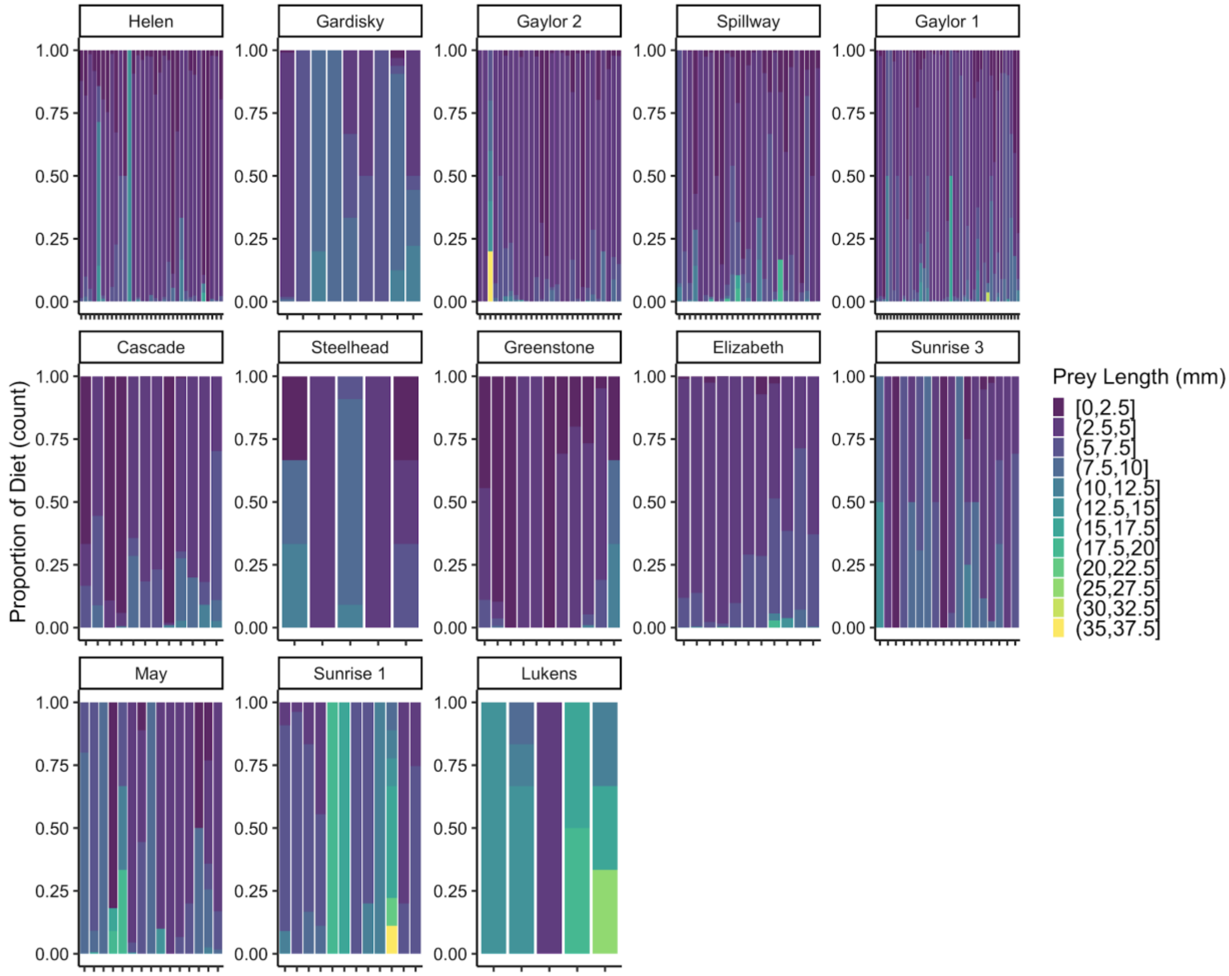

Figure A4. The prey length distribution of Brook Trout diets for each lake, arranged in descending order of elevation. Each vertical bar represents an individual fish.
